# ID1 regulates U87 human cell proliferation and invasion

**DOI:** 10.3892/ol.2013.1507

**Published:** 2013-08-01

**Authors:** PIN GUO, JIN LAN, JIANWEI GE, QING MAO, YONGMING QIU

**Affiliations:** 1 Department of Neurosurgery, Ren Ji Hospital, Shanghai Jiao Tong University School of Medicine, Shanghai 200127, P.R. China; 2 Shanghai Institute of Head Trauma, Shanghai 200127, P.R. China

**Keywords:** ID1, glioblastoma cell, invasion, proliferation

## Abstract

Despite therapeutic advances, the prognosis of patients diagnosed with malignant glioma has not improved in recent years. In particular, the molecular mechanisms that mediate glioma invasion remain poorly understood. The importance of ID1 in promoting tumor invasion and metastasis has recently emerged and a role for ID1 as a possible molecular marker of tumor aggressiveness has been proposed. To investigate the biological function of ID1 in glioblastomas, ID1-silenced U87 glioblastoma multiforme (GBM) cells were constructed using a small hairpin RNA (shRNA) sequence. The effect of the knockdown of ID1 on proliferation and invasion in these cells was analyzed using the 5-bromo-2′-deoxy-uridine cell proliferation, Transwell invasion, scratch and cell adhesion assays. Compared with the controls, the U87 cells expressing ID1-shRNA exhibited a significantly decreased proliferation and invasion capacity (P<0.05), as well as increased cell adhesion. Furthermore, silencing ID1 reduced the expression of c-Myc, cyclin D1 and β-catenin, while increasing E-cadherin expression in U87 cells. This study showed that ID1 regulates the metastatic potential of GBM cells by controlling the epithelial-mesenchymal transition. Therefore, ID1 is a potential prognostic indicator and therapeutic target in glioblastomas.

## Introduction

Despite advances in glioma treatments, including neurosurgery, radiotherapy and chemotherapy, the prognosis of patients diagnosed with malignant glioma has not improved in recent years (1–4). The poor prognosis of glioblastoma multiforme (GBM) is mainly due to tumor cell invasion of the brain tissue beyond the resected areas (5–7). Moreover, brain tumors are resistant to current therapies (6).

Glioma invasion is a complex cellular phenomenon that involves changes in the intracellular and extracellular biomechanical systems (4). Although our understanding of glioma oncogenesis is steadily improving, the molecular mechanisms that mediate glioma invasion remain poorly understood (8). There have been numerous studies describing the extracellular factors involved in glioma cell invasion, including β-catenin, c-Myc and cyclin D1 (9–11). However, the intracellular and molecular mechanisms that mediate glioma invasion require further elucidation in order to identify new drug targets.

ID proteins (inhibitors of DNA binding/differentiation) are helix-loop-helix transcription factors (12,13). The reactivation of the ID proteins, particularly ID1, promotes the development of several tumor types, including high-grade astrocytoma, prostate and breast cancers and non-small cell lung carcinoma (14–17). ID1 controls the expression of a large number of genes that mediate important cellular processes by inhibiting the activity of bHLH proteins (18–20). The main role of ID1 is to inhibit cell differentiation (18). In addition, loss of differentiation, unrestricted proliferation and increased cell invasion are hallmarks of malignancy. By maintaining an immature phenotype, ID1 enhances cell proliferation and invasion. Therefore, ID1 overexpression may induce invasion in several cancer types (21–23). The importance of ID1 in promoting tumor invasion and metastasis has emerged and a role as a possible molecular marker of tumor aggressiveness has been proposed (22). However, the role of ID1 in GBM is poorly understood.

In the present study, ID1-small hairpin RNA (shRNA)-expressing U87 cells and controls were constructed, and Transwell invasion and scratch assays were performed to analyze the effect of the knockdown of ID1 on cell invasion.

## Materials and methods

### Cell culture

The U87 human glioma cell line was obtained from the American Type Culture Collection (Manassas, VA, USA). The cells were grown in Dulbecco’s modified Eagle’s medium (DMEM; Gibco BRL, Gaithersburg, MD, USA) supplemented with 10% fetal bovine serum (FBS) at 37°C in a humidified atmosphere containing 5% CO_2_.

### qPCR

The U87 cells were washed three times with ice-cold phosphate-buffered saline (PBS) and total RNA was extracted using TRIzol (Invitrogen, Carlsbad, CA, USA). An equal amount of total RNA was used for first-strand cDNA synthesis using the oligo-dT primer and M-myeloblastosis virus reverse transcriptase XL (Promega, Madison, WI, USA) in a reaction volume of 25 μl, according to the manufacturer’s instructions. Synthesized first-strand cDNA (2 μl) was used for each PCR reaction.

qPCR experiments were performed using the SYBR Green PCR Master Mix (Applied Biosystems, Foster City, CA, USA). The PCR products were subjected to melting curve analysis to exclude the synthesis of non-specific products. The Ct value was quantified using a standard curve for the specific gene and relatively quantified using GAPDH as an internal reference control. The Ct value was then normalized to the average expression levels of undifferentiated samples, calculated according to the 2^-ΔΔCt^ method. All experiments were performed in triplicate.

### Western blotting

Cellular proteins (30 μg) were subjected to 12% SDS polyacrylamide gel electrophoresis (SDS-PAGE) and transferred onto Hybond ECL nitrocellulose membranes (Amersham, Piscataway, NJ, USA). Subsequent to being washed with 0.1% TBS-T, the membranes were blocked in 5% skimmed milk in TBS-T for 1 h at room temperature, then incubated with the appropriate antibody [1/500 dilution, ID1 antibody sc-488 (Santa Cruz Biotechnology, Inc., Santa Cruz, CA, USA); 1/1,000 dilution β-actin antibody (Abcam, London, England); 1/1,000 dilution, cyclin D1 antibody (Cell Signaling Technology, Danvers, MA, USA); 1/1,000 dilution, c-Myc antibody (Cell Signaling Technology); 1/1,000 dilution, β-catenin antibody (Abcam); 1/500 dilution, E-cadherin antibody (Cell Signaling Technology)] diluted in the same buffer overnight at 4°C. Subsequent to being washed with 0.1% TBS-T, the membranes were incubated with horseradish peroxidase-conjugated anti-rabbit or anti-mouse antibody, as appropriate (1/10,000 dilution; Sigma, St. Louis, MO, USA), for 2 h at room temperature. After washing with 0.1% TBS-T, specific protein bands were detected using western blotting detection reagents (Odyssey; Licor, Lincoln, NE, USA).

### Construction of U87 cells

The pGIPZ expression vector (Thermo Scientific, Waltham, MA, USA) carrying the ID1-shRNA coding sequence (shRNA sequence, TCGGAATCCGAAGTTGGAA) and the control empty vector were transfected into the U87 cells using Lipofectamine 2000 (Invitrogen), according to the manufacturer’s instructions. Two days after transfection, 800 μg/ml puromycin (Sigma) was added to the growth medium for the selection of stable ID1-shRNA-expressing cells. Colonies expressing marked green fluorescence were selected for further studies.

### BrdU proliferation of cells

Cell proliferation was assessed by 5-bromo-2′-deoxy-uridine (BrdU) incorporation using a Cell Proliferation ELISA, BrdU kit (Roche Applied Science, Indianapolis, IN, USA) according to the manufacturer’s instructions. The cells were seeded onto a 96-well plate at a density of 1×10^5^ cells/well in 100 μl culture medium and incubated at 37°C for 6, 12, 24 or 48 h. BrdU labeling solution was then added to a final concentration of 10 μM and the cells were incubated for an additional 2–4 h at 37°C. The medium was then removed and FixDenat (200 μl/well) was added to the cells and incubated for 30 min at 25°C. The FixDenat solution was then completely removed and 100 μl/well anti-BrdU-POD working solution was added and incubated for 90 min at 25°C The antibody conjugate was then removed by flicking and the wells were washed three times with 200 μl/well washing solution. Substrate solution (100 μl/well) was added and the cells were incubated at 25°C until color development was sufficient for photometric detection (after 6, 12, 24 and 48 h). The absorbance [optical density (OD)] at 450 nm was measured using a microplate reader.

### Cell adhesion assay

A 96-well plate was coated with Matrigel and incubated at 37°C for 1 h. The U87 cells were then plated at 5×10^4^ cells/well in serum-free MEM and the plate was incubated for 30 min at 37°C, followed by a gentle rinse with PBS to remove non-adherent cells. The cells were then fixed for 20 min with 3.5% formalin, stained with 0.5% crystal violet for 1 h and rinsed twice with PBS. The absorbance of the test samples and blank controls was measured at 594 nm using a microplate reader. The OD value of each test sample was designated the measured value and that of the blank was designated the blank value. The final value = measured value - blank value.

### Transwell invasion assay

Matrigel (25 mg reconstituted basement membrane) was added onto a polyvinylpyrrolidone-free polycarbonate filter (Nuclepore; Whatman, Maidstone, UK) and dried. The cells were harvested following brief exposure to 1 mM EDTA, then washed with DMEM containing 0.1% bovine serum albumin and added to Boyden chambers (2×10^5^ cells/chamber). The chambers were incubated for 24 h at 37°C. The cells that traversed the Matrigel layer and became attached to the filter were stained using a Diff-Quik kit (Dade Diagnostics, Aguada, PR, USA) and five randomized fields were counted. The mean ± SE was calculated for three independent experiments.

### Scratch assay

The cells were cultured to 90% confluency in six-well plates, then a thin scratch (wound) was made in the central area using a 10-ml pipette tip. Detached and damaged cells were carefully removed with PBS and the medium was replaced with serum-free medium. Wound closure was observed by light microscopy and images were captured at the indicated time points.

### Immunofluorescence

The cells were cultured on glass coverslips in 35-mm diameter dishes, washed with PBS and fixed with 4% p-formaldehyde for 15 min. Subsequent to being washed with PBS, the cells were incubated with 0.1% Triton X-100 for 25 min, washed three times with PBS and blocked with 10% goat serum for 30 min at room temperature. The cells were then incubated with an anti-actin antibody (1/100; Abcam) in a humidified chamber either overnight at 4°C or for 2 h at room temperature, followed by incubation with Alexa 488-conjugated goat anti-mouse antibody (1/200; Molecular Probes, Eugene, OR, USA) for 1 h at room temperature in a humidified chamber. DAPI staining was performed to identify the cell nuclei, and the cells were observed using a confocal Lasersharp 2000 version 5.1 microscope (Carl Zeiss, Jena, Germany) equipped with a Plan-Apochromat 63x/1.40 oil objective lens. Confocal images were acquired using LSM 5102.3 software (Alexa 488 emission was at 519 nm and Alexa 568 emission was at 603 nm).

### Statistical analysis

Data are represented as the mean ± SE from at least three independent experiments. All data were analyzed using an independent samples t-test using GraphPad Prism 5 software (San Diego, CA, USA). P<0.05 were considered to indicate a statistically significant difference.

## Results

### ID1-knockdown inhibits the proliferation of U87 cells

Our previous study showed that ID1 is highly expressed in several GBM cell lines, including A172, T98g, U251 and U87; of these, the U87 cells have the highest level of ID1 expression. Therefore, ID1 was knocked down in the U87 cells using ID1-shRNA expression and the changes in cell proliferation and invasion were measured between the ID1-silenced and control cells transfected with empty vector. It appears that ID1 expression was reduced in the U87 cells following shRNA-ID1 transfection (24).

**Figure 1:**
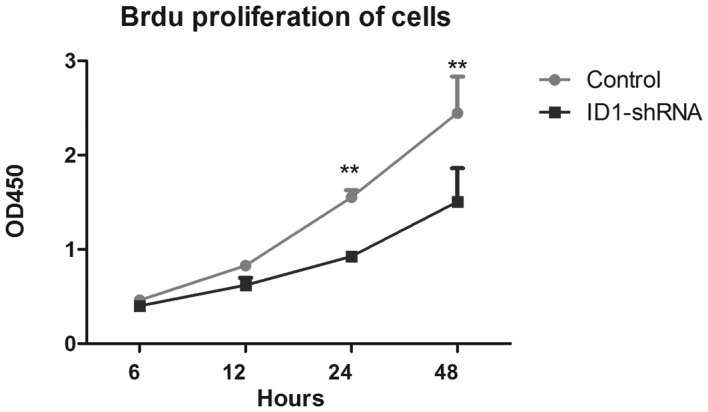
ID1-knockdown inhibits U87 cell proliferation. The numbers of ID1-shRNA-expressing and control U87 cells were compared at 6, 12, 24 and 48 h post-incubation with BrdU. At 24 and 48 h, the control group cells had higher OD values (^**^P<0.01, vs. ID1-shRNA). shRNA, small hairpin RNA; OD, optical density.

Cell proliferation is necessary for the development of all types of cancer, including gliomas. A previous study demonstrated that ID1 promotes tumor progression, although there is no direct evidence that ID1 is involved in GBM cell proliferation (25). The BrdU cell proliferation assay was therefore used to analyze the role of ID1 in GBM cell proliferation. As shown in Fig. 1, there were significant differences in cell proliferation between the ID1-silenced and control cells at the 24- and 48-h time points; decreased ID1 expression correlated with a reduction in U87 cell proliferation. These data suggest that ID1 has a role in blocking aberrant U87 cell proliferation.

### ID1-knockdown reduces the invasiveness of U87 cells

**Figure 2:**
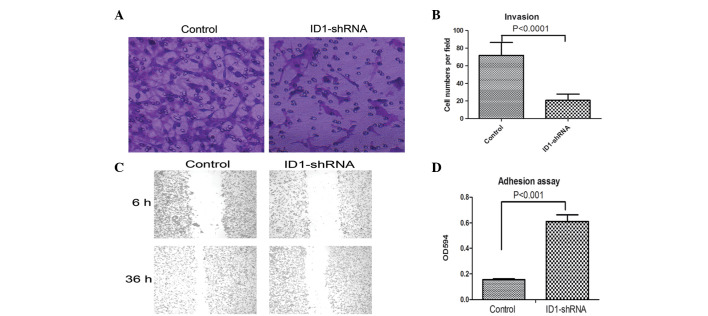
ID1-knockdown inhibits U87 cell invasion. (A) Cells were stained with crystal violet and then observed by light microscopy (magnification, ×100).(A and B) Transwell invasion assay showing differences in invasiveness between ID1-shRNA-expressing and control U87 cells. (C) Scratch assay comparing the wound healing properties of ID1-shRNA-expressing and control U87 cells (magnification, ×20). (D) Adhesion assay comparing the adhesion ability of ID1-shRNA-expressing and control U87 cells.

Transwell invasion and scratch assays were used to measure the rate of invasion of the ID1-silenced and control U87 cells. The Transwell invasion assay showed that fewer ID1-shRNA-expressing U87 cells passed through a polycarbonate membrane compared with the controls (P<0.0001; Fig. 2A and B). The scratch assay showed that the there was no significant difference in wound healing between the two groups of cells after 6 h. However, after 36 h, there was almost complete wound healing in the control cells, but not in the U87 cells (Fig. 2C). This result is consistent with the result of the Transwell invasion assay, i.e., that wound healing and migration are inhibited by reduced ID1 expression. These data suggest that ID1-knockdown blocks U87 cell invasion.

### ID1-knockdown enhances U87 cell adhesion

Reduced adhesion is necessary for the increase in cell mobility and invasion capacity. A cell adhesion assay was used to compare the adhesive properties of ID1-silenced and control cells. As shown in Fig. 2D, the U87 cells with ID1-knockdown were more adhesive compared with the control cells, i.e., a change in ID1 expression led to a change in cell adhesion. Compared with the controls, the ID1-silenced U87 cells showed more marked adhesive properties. This is consistent with higher levels of cell invasion in normal U87 cells.

### Effects of ID1-knockdown on U87 morphology and cytoskeleton

**Figure 3:**
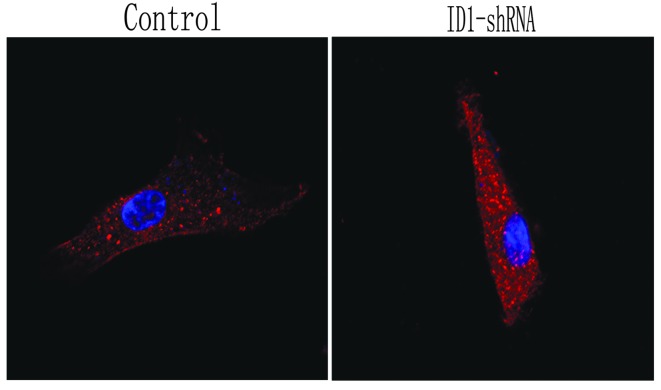
ID1-knockdown induces alterations in the actin cytoskeleton of U87 cells. ID1-shRNA-expressing and control U87 cells were stained with an anti-actin antibody and observed by confocal microscopy (magnification, ×400).

In order to observe the morphological and cytoskeletal changes in the U87 cells following the silencing of ID1, actin filaments were observed by immunofluorescence and confocal scanning laser microscopy in the treated and control cells. In the control cells, actin was localized and highly expressed on pseudopodia around the nucleus and on stress fibers. However, the U87 cells expressing ID1-shRNA became flatter and smaller, with shorter lamellipodia compared with the controls (Fig. 3). Thus, cytoskeletal changes due to ID1 downregulation are associated with altered tumor cell invasion properties. These data support the hypothesis that U87 cell invasion is reduced following the knockdown of ID1.

### ID1 regulates expression of β-catenin, cyclin D1, c-Myc and E-cadherin

**Figure 4:**
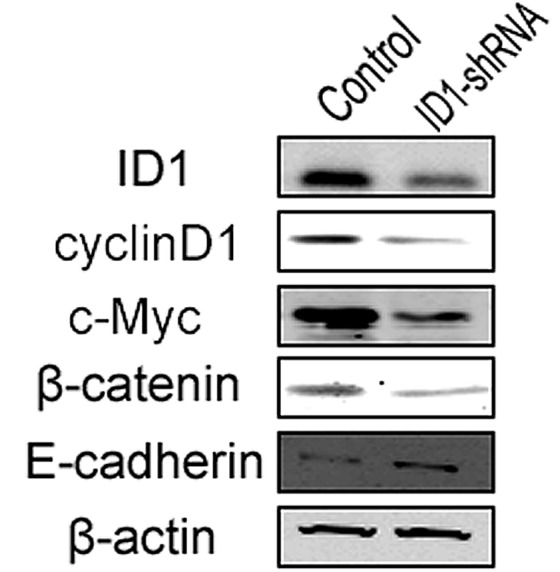
ID1 regulates factors that are involved in the proliferation and invasion of U87 cells. The expression levels of CyclinD1, C-myc and β-catenin were reduced in ID1-shRNA-expressing U87 cells, while E-cadherin expression was increased compared with the controls.

Several signaling molecules have been reported to contribute to the increased invasion and proliferation properties of certain types of cancer; these include c-Myc, cyclin D1 and β-catenin. For example, the β-catenin pathway is critical in glioma tumor invasion (10), while c-Myc and cyclin D1 are also involved in several pathways that promote GBM invasion. Therefore, the expression levels of these proteins were measured in the ID1-silenced and control cells in order to determine whether protein levels correlate with invasiveness. In the U87 cells, ID1-knockdown led to the reduced expression of c-Myc, cyclin D1 and β-catenin (Fig. 4). The epithelial-mesenchymal transition (EMT) increases the invasive capacity of tumor cells (26). As previously reported, the level of E-cadherin expression is a critical marker of EMT (27). The present data showed that E-cadherin expression was increased following ID1-knockdown, i.e., a reduction in ID1 expression inhibits EMT and thus invasiveness in U87 cells.

## Discussion

GBM is the most common type of central nervous system malignancy and the prognosis of patients with GBM is not improved by standard treatments (1,3). The majority of GBMs are poorly differentiated; this is linked to tumor aggression and lethality (28). Malignant glioma cell proliferation and invasion are key stages in cancer progression that affect patient mortality (29). Clinically, there are limited therapeutic interventions for malignant glioma. Therefore, more research into the mechanisms of GBM invasion is essential for the development of a curative therapy.

ID1, an inhibitor of basic helix-loop-helix transcription factors, has been shown to be a key regulator of a number of steps in cancer progression (12,13). Moreover, ID1 inhibits cell differentiation and promotes invasion in several types of malignant cancers, including breast and prostate cancers and non-small cell lung carcinoma (14,16,23). Generally, ID1 contributes to tumorigenesis by inhibiting cell differentiation, stimulating proliferation, enhancing invasion and facilitating tumor neoangiogenesis (30,31). Perk *et al*, however, suggested that ID1 function may depend on cell type (13). Meng *et al* demonstrated that ID1 induces differentiation in mouse embryonic carcinoma P19CL6 cells (32). Furthermore, Geng *et al* reported that ID1 enhances docetaxel cytotoxicity in prostate cancer cells through p21 inhibition and suggested that ID1 is a novel prognostic marker and therapeutic target in prostate cancer chemotherapy (33). There have been several studies of ID1 in glioma. Vandeputte *et al* reported that ID expression is lower in low-grade astrocytoma compared to high-grade astrocytoma and therefore inferred that ID1 expression levels are associated with the grade of glioma (17). Anido *et al* identified a cell population enriched with glioma-initiating cells (GICs) that express high levels of ID1 and suggested that high ID1 levels are associated with a poor prognosis in GBM patients (34). By contrast, Barrett *et al* reported that an improved prognosis is associated with higher ID1 expression in a preneural subgroup of GBM, even though ID1 overexpression correlates with increased self-renewal in GICs (35). These contradictory studies show that further investigations are required to determine the function of ID1 in GBM.

Since ID1 promotes cell proliferation and invasion, it has been proposed as an attractive target for cancer therapy. Therefore, in the present study, an ID1-shRNA transfection system was established for U87 cells to investigate the correlation between ID1 expression and biological outcome. Using Transwell invasion and scratch assays, it was observed that ID1-shRNA-expressing U87 cells have a poorer invasion ability. In addition, it was demonstrated that compared with ID1-silenced U87 cells, the controls had poorer adhesion properties. The effect of ID1 silencing on cell proliferation was also investigated, as this contributes to tumor invasion. The BrdU proliferation assay was used to analyze cell proliferation in the U87 cells and ID1-knockdown was observed to lead to decreased proliferation.

In general, cell movement requires morphological changes that involve breaking down and reforming cytoskeletal filaments (36). Therefore, to determine whether changes in ID1-mediated cell invasion correlate with cytoskeletal alterations, actin filaments were visualized by immunofluorescence and changes in cell shape were determined following ID1-knockdown. The ID1-silenced cells had fewer lamellipodia and became rounder and smaller compared with the control cells. Such changes in cell morphology are associated with increased invasiveness and may be a consequence of the dysregulation of ID1 signaling components mediating proliferation and invasion (36). Factors such as cyclin D1, c-Myc and β-catenin have been shown to promote GBM cell invasion. Therefore, the expression of these proteins was examined in the ID1-silenced and control U87 cells (37). These proteins were all observed to be downregulated in the ID1-shRNA-expressing U87 cells. By contrast, ID1-knockdown increased E-cadherin protein levels, which are considered to be a marker of EMT. ID1 silencing may inhibit the process of EMT, which is pivotal in cell invasion. The present results therefore suggest that ID1-knockdown may inhibit U87 glioma cell proliferation and invasion.

The present study demonstrated that the loss or inhibition of ID1 expression may be important in GBM cell proliferation and invasion. However, the specific mechanism through which ID1 regulates these GBM cell properties requires further research, which may lead to the identification of new strategies and therapeutic targets for glioma treatment.
